# Loss of Stromal Galectin-1 Enhances Multiple Myeloma Development: Emphasis on a Role in Osteoclasts

**DOI:** 10.3390/cancers11020261

**Published:** 2019-02-23

**Authors:** Joséphine Muller, Elodie Duray, Margaux Lejeune, Sophie Dubois, Erwan Plougonven, Angélique Léonard, Paola Storti, Nicola Giuliani, Martine Cohen-Solal, Ute Hempel, Victor L. Thijssen, Yves Beguin, Roy Heusschen, Jo Caers

**Affiliations:** 1 Laboratory of Hematology, GIGA-Research, University of Liège, 4000 Liège, Belgium; josephine.muller@skynet.be (J.M.); elodie.duray@doct.uliege.be (E.D.); margauxlejeune28@gmail.com (M.L.); sophie.dubois@chuliege.be (S.D.); yves.beguin@chuliege.be (Y.B.); roy_heusschen@hotmail.com (R.H.); 2 Department of Chemical Engineering, University of Liège, 4000 Liège, Belgium; eplougonven@uliege.be (E.P.); a.leonard@uliege.be (A.L.); 3 Department of Medicine and Surgery, University of Parma, 43121 Parma, Italy; paola.storti@unipr.it (P.S.); nicola.giuliani@unipr.it (N.G.); 4 Department of Rheumatology, Hôpital Lariboisière, INSERM-UMR 606, Université Paris Diderot, 75010 Paris, France; martine.cohen-solal@inserm.fr; 5 Institute of Physiological Chemistry, Faculty of Medicine Carl Gustav Carus, Technische Universität Dresden, 01307 Dresden, Germany; hempel-u@mail.zih.tu-dresden.de; 6 Amsterdam UMC, Vrije Universiteit Amsterdam, Medical Oncology & Radiation Oncology, 1081 Amsterdam, The Netherlands; v.thijssen@vumc.nl; 7 Department of Medicine, Division of Hematology, University and CHU of Liège, 4000 Liège, Belgium

**Keywords:** multiple myeloma, osteolytic disease, galectin-1, osteoclasts, tumour microenvironment

## Abstract

Multiple myeloma osteolytic disease is caused by an uncoupled bone-remodelling process with an increased osteoclast activity. Disease development relies on interactions between myeloma cells and bone marrow stromal cells. Recent findings suggest a role for glycan-binding proteins in myeloma microenvironment. Here, we investigated lectins involved in osteoclastogenesis and their role in myeloma bone disease. Microarray data analysis showed a lower expression of galectin-1 (gal-1) in mature osteoclasts compared to monocytic progenitor cells, confirmed at the RNA and protein levels in osteoclast cultures. Confocal microscopy showed that gal-1 localised predominantly in the sealing zone of mature osteoclasts. Although equal differentiated-osteoclast numbers, gal-1^−/−^ osteoclasts showed a higher resorption activity compared to wild-type controls. Micro-computed tomography showed an aberrant bone phenotype with decreased bone densities in gal-1^−/−^ mice. In vivo, tumour progression was faster in gal-1^−/−^ mice and associated with a marked bone loss. Additionally, myeloma cells were found to decrease gal-1 expression in osteoclasts. Our results demonstrate that galectin-1 regulates osteoclast activity with an increased resorption by gal-1^−/−^ osteoclasts and decreased bone densities in gal-1^−/−^ mice. We observed an enhanced tumour development in gal-1^−/−^ mice compared to wild-type mice, suggesting that galectin-1 has a functional role in stromal cells in myeloma microenvironment.

## 1. Introduction

Multiple myeloma (MM) is a haematological malignancy characterised by the clonal proliferation of plasma cells in the bone marrow. Multiple myeloma bone disease (MMBD) is a hallmark of MM and characterised by both reduced bone formation and increased bone resorption, the latter due to an activation of osteoclast activity. Interactions between myeloma cells and bone marrow stromal cells are essential for the development of MMBD [1]. Recent data support a role for the glycan-binding protein galectin-1 in both myeloma cells and in the MM microenvironment. Galectin-1 stimulates angiogenesis and supports tumour cell proliferation in MM [2]. In addition, it has been identified as an extracellular matrix (ECM)-associated protein that is more abundant in the MM microenvironment [3].

Galectin-1, a lectin with a broad range of biological activities [4,5,6,7], is differentially expressed by many cancer cells and is frequently found in the stroma surrounding tumour cells [8]. It belongs to the galectin protein family of which the members have a binding specificity for β-galactose-containing glycans. Galectins trigger intracellular signalling through crosslinking of cell surface glycoprotein receptors or glycolipids on the same cell, between cells and in cell-matrix interactions. They were shown to contribute to many hallmarks of cancer [9] and their dysfunction or altered expression has frequently been associated with cancer [10,11,12].

Despite the wide expression and pleiotropic roles of galectins in normal and cancerous tissues, their implication in bone cell function is only poorly understood. Given the emerging role of galectin-1 in MM biology, we explored the involvement of galectins in osteoclasts and the contribution of stromal galectin-1 to MM development.

## 2. Results

### 2.1. Galectin-1 Expression Decreases during Osteoclast Differentiation

**Figure 1:**
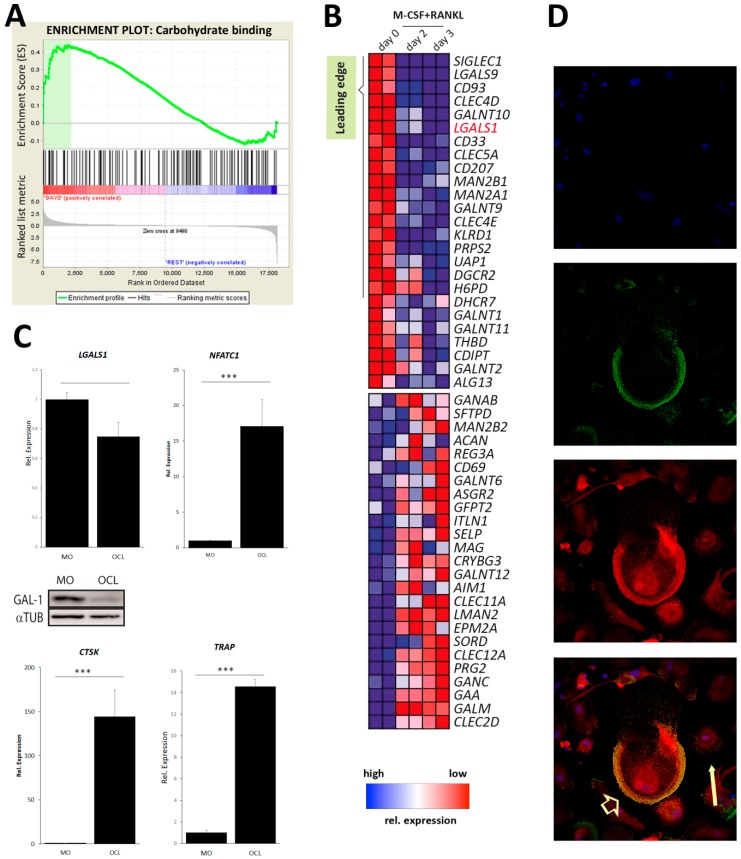
Galectin-1 expression decreases during osteoclast differentiation. (**A**) GSEA representing the distribution of the carbohydrate gene set in monocytes/macrophages versus osteoclasts (OCLs) and demonstrating that OCL precursors are higher enriched in carbohydrate binding proteins compared to mature OCLs. (**B**) Expression data set sorted by correlation with phenotype, the corresponding heat map, the gene tags and the leading-edge subset. Genes are ranked based on the correlation between their expression and the class distinction. (**C**) qPCR analyses of gal-1, including western blot analysis and OCL reference gene (NFATc1, cathepsin K, TRAP) expression in RAW-264.7-derived osteoclast cultures (MO: monocytes; OCL: osteoclasts; αTUB: α-tubulin). Data are representative of three (*n* = 3) biologically independent experiments and represented as mean +/− standard error. * *p* < 0.05; ** *p* < 0.01; *** *p* < 0.001. (**D**) Localization of gal-1 in mononuclear precursors (arrow) and mature OCLs (arrow head) (top to bottom: nucleus; actin, galectin-1, merge). (magnification: 60×) Representative images out of three (*n* = 3) independent experiments are shown.

MMBD is characterised by an increased osteoclast activity. Given the emerging role of lectins in osteoclast function, we performed a gene set enrichment analysis (GSEA) with a custom gene set for carbohydrate-binding proteins in publicly available microarray data of primary bone marrow monocyte-derived osteoclast differentiation. Carbohydrate-binding signatures were enriched in monocytes compared to mature osteoclasts (Figure 1A). The false-discovery rate was 0.09 and the normalised enrichment score was 1.45. A heat map of the top 25 positively and negatively correlated genes is shown as well as the leading edge of the GSEA (Figure 1B). The gene encoding galectin-1, that is, *LGALS1*, appeared in the leading edge as one of the core genes that account for the enrichment signal in monocytes. These microarray data were validated in RAW264.7-derived osteoclast cultures. This confirmed that galectin-1 mRNA and protein levels were decreased in mature osteoclasts compared to monocytes (Figure 1C; complete WB: Figure S3). Of note, markers of osteoclast maturation, that is, nuclear factor of activated T-cells 1 (NFATc1), cathepsin K (CTSK) and tartrate-resistant acid phosphatase (TRAP), all showed a significant increase in mRNA expression levels (Figure 1C). Further analysis of galectin-1 localization in mature osteoclasts revealed that, although galectin-1 levels markedly decreased in osteoclasts compared to monocytes, galectin-1 protein expression remained detectable in the sealing zone of mature osteoclasts (Figure 1D).

### 2.2. Loss of Galectin-1 Enhances Bone Matrix Resorption by Osteoclasts

**Figure 2:**
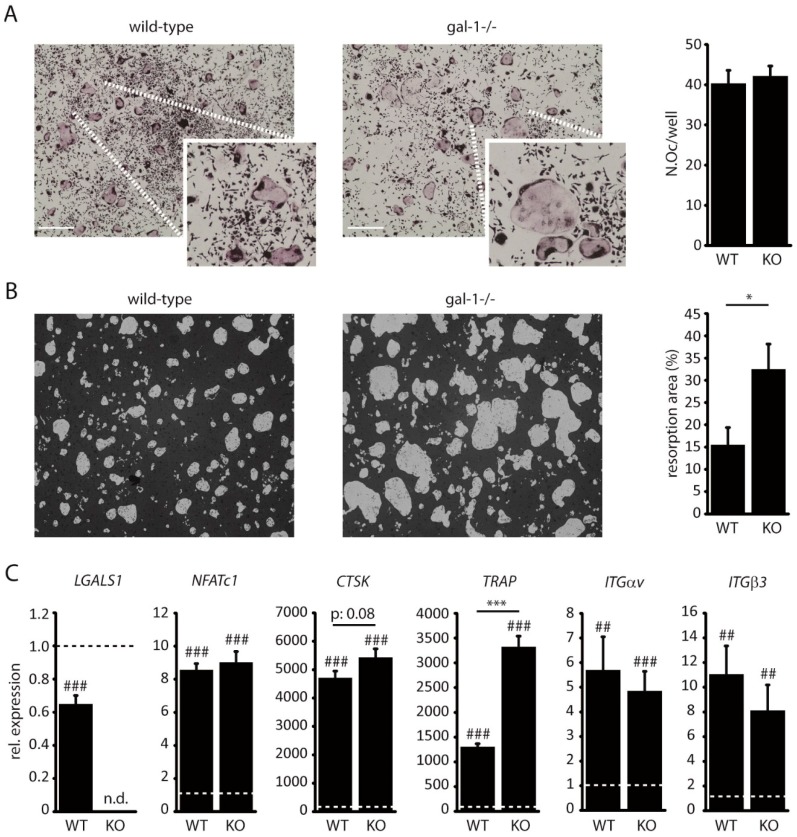
Loss of gal-1 enhances bone matrix resorption by osteoclasts. (**A**) Representative images of TRAP-stained primary (left) wild-type and (centre) gal-1^−/−^-derived osteoclast cultures. Quantification (right) of osteoclast number per well. (scale: 100 µm) (**B**) Resorbed matrix and quantification of the resorbed area. (**C**) Real-time PCR of osteoclast differentiation markers in mature osteoclasts derived from wild-type and gal-1^−/−^ mice versus monocyte cultures (dotted line) (n.d.: not detected). From left to right: galectin-1 (LGALS1), NFATc1, cathepsin K (CTSK), TRAP, Integrin αv (ITGαv) and integrin β3 (ITGβ3). Significance level versus monocyte cultures. All data are representative of three (*n* = 3) biologically independent experiments and represented as mean +/− standard error. ## *p* < 0.05; ### *p* < 0.001; * *p* < 0.05; *** *p* < 0.001.

In order to elucidate the functional role of galectin-1 in osteoclasts, we established primary osteoclast cultures from C57BL6 wild-type and C57BL6 gal-1^−/−^ mice. There was no difference in osteoclast differentiation between wild-type and gal-1^−/−^ cultures (Figure 2A). In contrast, the loss of galectin-1 resulted in a 2-fold increase in bone matrix resorption by osteoclasts (Figure 2B). Comparison of osteoclast marker gene expression between wild-type and gal-1^−/−^ osteoclasts revealed an increased TRAP expression (Figure 2C).

### 2.3. C57BL6 gal-1^−/−^ Mice Have a Decreased Bone Mass

**Figure 3:**
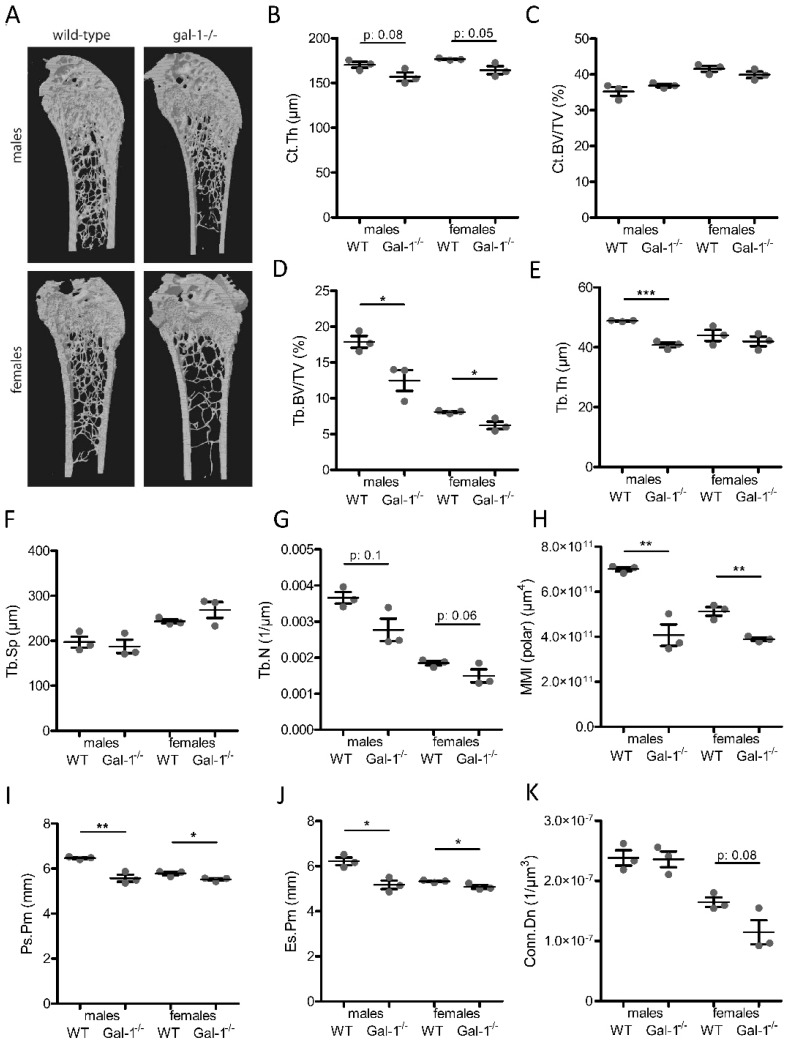
C57BL/6 gal-1^−/−^ have a decreased bone mass. (**A**) Representative 3D-reconstructions of distal femurs. CTAn analysis was performed and (**B**) cortical thickness (Ct;Th), (**C**) cortical bone volume (Ct.BV/TV), (**D**) trabecular bone volume (Tb.BV/TV), (**E**) trabecular thickness (Tb.Th), (**F**) trabecular separation (Tb.Sp), (**G**) trabecular number (Tb.N), (**H**) polar mean moment of inertia (MMI(polar)), (**I**) periosteal perimeter, (**J**) endosteal perimeter and (**K**) trabecular connective density (Conn.Dn) are reported here. Data shown are the mean +/− standard error of three mice and all results shown are representative of three biologically independent experiments. * *p* < 0.05; ** *p* < 0.01; *** *p* < 0.001.

To further explore the role of galectin-1 in bone turnover, we compared bones of C57BL6 wild-type with C57BL6 gal-1^−/−^ mice. Galectin-1^−/−^ tibias and femurs appeared macroscopically to be shorter and thinner compared to wild-type bones (data not shown). Subsequent X-ray micro-computed tomography (µCT) analyses on distal femurs confirmed that gal-1^−/−^ bones have a decreased cortical and trabecular bone mass compared to wild-type bones (Figure 3A). Of note, analysis was performed separately on males and females due to sex differences in skeletal mass and structure, as previously reported [13,14]. Cortical thickness (Ct.Th) was significantly reduced in gal-1^−/−^ bones (Figure 3B). Cortical bone volume (Ct.BV/TV) was not different (Figure 3C). Regarding trabecular bone, trabecular bone volume (Tb.BV/TV) was reduced in gal-1^−/−^ bone compared to wild-type bones (Figure 3D). This was most likely due to a decrease in trabecular thickness (Tb.Th) and trabecular number (Tb.N) (Figure 3E,G). Trabecular separation (Tb.Sp) was not different (Figure 3F). Additionally, a significant reduction in the polar mean moment of inertia (polarMMI) (Figure 3H) points a potential reduced cortical bone strength, although this assumption requires further mechanical properties testing for confirmation. Connectivity density (Conn.Dn) (Figure 3K) was only decreased in female mice. Additionally, periosteal perimeter (Ps.Pm) and endosteal perimeter (Es.Pm) were both significantly reduced in gal-1^−/−^ bones (Figure 3I,J). Collectively, these observations are indicative of an impaired bone development in gal-1^−/−^ mice as compared to wild-type animals.

### 2.4. Loss of Stromal Galectin-1 Enhances In Vivo Multiple Myeloma Development and Exacerbates Myeloma Bone Disease

**Figure 4:**
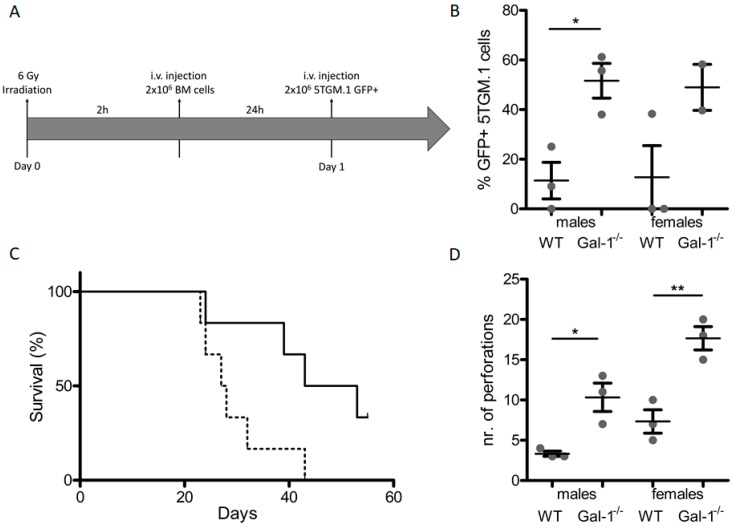
Loss of stromal gal-1 enhances myeloma growth. (**A**) MM C57BL/6 WT and gal-1^−/−^ model. Mice were irradiated at 6Gy and subsequently received syngeneic bone marrow (BM) cells. MM cells (5TGM.1 GFP+) were injected 24 h post-transplantation. (**B**) Percentage of 5TGM.1 GFP+ bone marrow infiltration. (**C**) Survival curve (solid line: wild-type; dotted line: gal-1^−/−^). (**D**) Bone µCT analysis of WT and gal-1^−/−^ MM-bearing mice, showing the number of cortical perforations. Data shown are the mean +/− standard error of three mice and are representative of three biologically independent experiments. * *p* < 0.05; ** *p* < 0.01.

We used a transplantation-based approach that allows for engraftment of 5TGM.1 MM cells in the C57BL/6 background to assess the role of stromal galectin-1 in MM development (Figure 4A). MM development was enhanced in C57BL/6 gal-1^−/−^ mice compared to wild-type controls (Figure 4B), with a corresponding early paraplegic development and a decrease in survival of these mice (Figure 4C). C57BL/6 wild-type mice transplanted with 5TGM.1 MM cells displayed MMBD, which was further exacerbated in MM-bearing gal-1^−/−^ mice, as reflected by an increase in the number of cortical perforations, a defining characteristic of MMBD. Furthermore, cortical thickness and trabecular parameters were differently affected in MM-bearing wild-type versus gal-1^−/−^ mice (Supplementary Figure S1). To assess the contribution of increased bone turnover to the enhanced MM development, we treated MM-bearing mice with the bisphosphonate pamidronate that inhibits osteoclast activity but has no antitumor activity. Indeed, MTT assays confirmed that bisphosphonate did not affect 5TGM.1 cell proliferation. Regarding MM development, pamidronate treatment did not affect the survival of gal-1^−/−^ mice nor bone marrow infiltration by 5TGM.1 cells (Supplementary Figure S2).

### 2.5. Multiple Myeloma Cells Induce Decreased Galectin-1 Levels in Mature Osteoclasts In Vitro and in Bone Marrow Stromal Cells from Patients with Bone Disease

**Figure 5:**
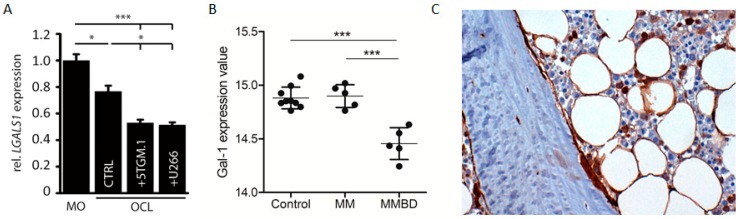
Myeloma cells induce decreased gal-1 levels in mature osteoclasts. (**A**) Relative galectin-1 mRNA expression to monocytes cultures in osteoclast culture (CTL), osteoclast-5TGM-1 and -U266 transwell co-cultures. (MO: monocytes; OCL: osteoclasts) (**B**) Gal-1 mRNA expression in primary BMSCs (publicly available dataset: GSE85837) of donors without malignant BM involvement (control; *n* = 9), MM patients without bone involvement (MM; *n* = 5) and MM patients with bone disease (MMBD; *n* = 5). (**C**) Gal-1 protein was evaluated by immunohistochemistry in fixed bone biopsies obtained from 15 MM, 9 asymptomatic MM and 10 MGUS patients. The photo shows gal-1 protein immunostaining of osteomedullary biopsies from one representative asymptomatic MM patient Magnification 20× (Materials & Methods: [2]). Data in Figure 5A are representative of three (*n* = 3) biologically independent experiment. Data in Figure 5B are representative of one analysis (*n* = 9 control; *n* = 5 MM; *n* = 5 MMBD). All results are represented as mean +/− standard error. * *p* < 0.05; *** *p* < 0.001.

Finally, we assessed the impact of myeloma cells on galectin-1 expression during osteoclastogenesis. We observed that human U266 and murine 5TGM.1 cell lines further decreased the galectin-1 expression levels in mature osteoclasts when co-cultured in a transwell system (Figure 5A). Clinically, gal-1 levels appear lower in primary bone marrow stromal cells (BMSCs) from MM patients with bone disease (MMBD) compared to primary BMSCs from donors without malignant bone marrow involvement (control) or MM patients without myeloma bone disease (MM) (Figure 5B). Additionally, the expression of gal-1 in the matrix and bone cells of bone marrow sections from myeloma patients (15 MM, 9 asymptomatic MM and 10 benign precursor disease MGUS (monoclonal gammopathy of undetermined significance)) was investigated by immunohistochemistry. Analysis of sections showed that galectin-1 is mainly expressed by osteoblasts, independently of the stage of disease. Osteocytes were also found to express gal-1 while the bone matrix was negative. Evaluation of osteoclasts could not be performed because of the lack of TRAP counterstaining.

## 3. Discussion

Glycorecognition systems are involved in numerous processes, such as mediation of inflammation and immune response, protein trafficking, cell adhesion and migration, cancer and cytotoxicity [15,16,17]. Changes in the cellular glycosylation are associated with malignant transformation of cancer cells, tumour progression and metastasis formation [18]. Interactions between cancer cells and the tumour microenvironment rely on glycans, which are also implicated in the bone microenvironment and homeostasis [19].

MMBD is one of the major features of MM and is a major cause of morbidity and mortality. Myeloma cells stimulate osteoclast activity and bone resorption while inhibiting osteoblast function. In the current study, we set out to study the role of glycan-binding proteins in stromal cells, with a focus on osteoclasts.

Analysis of publicly available expression data identified galectin-1 as a potential regulator of osteoclast biology. Our functional data suggest that galectin-1 is involved in osteoclast activity and that loss of galectin-1 in the MM microenvironment increases cortical perforations and potentiates MM development. Of note, other galectin family members have been implicated in osteoclast biology, such as galectin-3, which in vitro interferes with RANKL signalling on osteoclasts, reducing their differentiation and galectin-3 deficient mice displaying increased osteoclast activity [19,20]. Moreover, galectin-9, secreted by the osteoclasts, is significantly upregulated during osteoclastogenesis and is involved in the immune suppressive microenvironment in MM [21].

During osteoclast maturation, GSEA and osteoclast cultures showed that galectin-1 expression decreases while confocal microscopy demonstrated a localization of galectin-1 at the sealing zone in mature osteoclasts. The sealing zone, that defines the resorption area of the bone, consists of a large actin ring and adhesion molecules, such as integrins. Osteoclast express α_v_β_3,_ α_2_β_1_ and α_v_β_1_ integrins. Interestingly, the β1 integrin is a galectin-1 receptor [22]. Follow-up studies are necessary to explore potential interactions. Moreover, studies have reported that galectin-1 is implicated in myoblast and trophoblast cell fusion [23,24,25,26], the latter notably by interacting with β1 integrin [27]. All could imply a role of gal-1 in the osteoclast fusion or in the formation/adhesion of the sealing zone and the restricted acid resorption zone.

Basic macroscopic analysis of gal-1 deficient mice indicated shorter bones, although further measurements are required, with a decreased bone mass as determined by µCT. Poirier and Robertson, who developed the galectin-1 knockout model, initially indicated no change in development or obvious phenotypic differences as compared to wild-type animals, suggesting that gal-1 functions could be largely compensated for in vivo. [28]. Additionally, a study focusing on the phenotype of mutant mouse strains with gene-targeted defects in glycosyltransferases or glycan-binding proteins [29] did not report any bone phenotype in gal-1^−/−^, although impacts on B- and T-cell proliferation and T-independent response as well as changes in Sca-1 (Ly-6A) were mentioned. These changes were not previously detected, probably because no macroscopic differences are observed and because of a lack of interest. Similar subtle phenotypic abnormalities have been described during embryologic development [30].

Our results indicate that myeloma-bearing mice lacking gal-1 expression have higher bone marrow infiltration associated with an enhanced tumour development, an early appearance of symptoms and a shorter survival. This observation is in line with a recent study showing that extracellular matrix remodelling occurred during disease progression and that galectin-1, as part of the ECM proteins, had prognostic relevance for MM patient overall survival [3]. However, galectin-1 is known to exert pleiotropic roles in the tumour microenvironment. Previous studies focused on the contribution of stromal galectin-1 in angiogenesis, T-cell responses and mesenchymal stem cells [7].

Since bone homeostasis was unexplored in gal-1^−/−^ mice and bone resorption contributes to myeloma development, we focused our work on osteoclast activation and tumour progression. However, the effects observed in the MM-bearing gal-1^−/−^ mice might not only be due to aberrant bone cells and therefore further studies on, for example, inflammatory cells are needed. Moreover, mice were irradiated, followed by stem cell transplantation and tumour inoculation. This was done to allow tumour engraftment in C57BL/6 mice, while these tumours only thrive in C57BL/KaLwRij. Irradiation and stem cell transplantation may induce other changes in the tumour environment that support MM growth [31]. In addition, irradiation may contribute to the changes observed in the trabecular bone parameters, which can already appear at one week after irradiation [32]. We believe that the experimental procedures cannot explain the marked differences in cortical perforations between gal-1 C57BL/6 mice. Backcrossing gal-1^−/−^ mice in RAG^−/−^ mice, which was previously used to point out the contribution of stromal MMP-7 to the osteolytic process [33], also results in immunodeficient mice lacking B and T-cell responses that may influence tumour take and development. The ultimate proof would be the selective knock-out of gal-1 in osteoclasts or its precursor cells using the cre-lox system [34].

Myeloma-bearing gal-1^−/−^ mice showed increased myeloma bone disease compared to wild-type mice. Equally, we observed a decrease of gal-1 expression in mature osteoclasts co-cultured with myeloma cells and, clinically, a decrease of gal-1 level in bone marrow stromal cells from MM patients with bone disease. MMBD in gal-1^−/−^ mice was marked by an increase in the number of cortical perforations, osteolytic lesions being the most dramatic manifestation of bone loss in myeloma bone disease. Interfering osteoclast activity with pamidronate had no impact on myeloma development, as reported by previous studies [35,36], supporting the assumption that MMBD and MM growth are not necessarily interconnected in murine models.

Osteoclast-myeloma cell co-cultures further reduced gal-1 expression during differentiation. Osteoclasts-myeloma cell interactions occur through cell-cell contact that involve α_4_β_1_ (VLA-4) and α_v_β_3_ integrins leading to enhanced MM cell growth and survival [37]. However, we observed a decrease in galectin-1 expression in both direct and indirect transwell co-cultures, suggesting that a secreted factor could be responsible for the potent reduction of galectin-1 expression in osteoclasts. Identifying this factor could become of therapeutic value.

Regarding the clinical perspective, our data highlight the great importance of lectin-glycan interactions in cancer development. Standard therapeutic strategies for bone metastases and multiple myeloma mainly rely on bisphosphonates. Unfortunately, their use is linked to side effects, such as the development of atypical fractures. Identifying new interfering molecules, such as galectin-1 or mediators of osteoclast function, is of great interest for new precise and targeted therapy.

## 4. Materials and Methods

### 4.1. Gene Set Enrichment Analysis

We performed a gene set enrichment analysis (GSEA) to determine whether carbohydrate-binding signature gene sets were enriched in primary murine bone marrow derived monocytes (BMM) versus BMM stimulated with M-CSF and RANKL during 0, 2 and 3 days. The data discussed in this publication have been deposited in NCBI’s Gene Expression Omnibus [38] and are accessible through GEO Series accession number GSE57468 [39]. We generated a custom gene set to test in this microarray dataset consisting of all genes annotated by gene ontology term GO:0030246 (carbohydrate binding). GSEA was performed according to the developer’s protocol (www.broad.mit.edu/gsea/) [40]. The expression of galectins and osteoclast reporter genes was analysed in GSE57468 by GEO2R analysis. FDR scores <0.25 were considered significant [40].

### 4.2. Galectin Expression Analysis in Patients

GEO2R analysis of publicly available microarray data was carried out to examine the expression of galectin-1 in primary bone marrow stromal cells from patients with and without osteolytic bone lesions (GSE85837).

### 4.3. Cells and Culture Conditions

RAW264.7 and 5TGM.1 GFP+ cells were cultured in Dulbecco’s Modified Eagle’s Medium (DMEM) (Lonza, Belgium) supplemented with 10% foetal bovine serum (FBS; Sigma-Aldrich, St-Louis, MO, USA), 2 mM L-glutamine (Lonza, Verviers, Belgium) and 100 U/mL penicillin-streptomycin (P/S; Lonza). All cell lines were cultivated at 37 °C in 5% CO_2_ humidity.

### 4.4. Proliferation Assay

Cell proliferation and viability was assessed with the cell proliferation kit I (Roche, Basel, Switzerland) according to the supplier’s protocol. In short, cultures in 96 well plates were incubated with 10 µL MTT labelling reagent for 4 h at 37 °C. Next, 100 µL solubilization reagent was added to each well and incubated overnight at 37 °C. The next day, absorbance was measured at 570 nm on a Wallac 1420 Victor2 microplate reader (Perkin Elmer, Waltham, MA, USA).

### 4.5. Osteoclast Differentiation and Activity Assay

Murine primary (twelve-week old mice of both sexes) and RAW264.7-derived osteoclast cultures were established as described previously [41]. Of note, the osteoclast differentiation medium for both primary and cell line-derived cells was α-MEM supplemented with 10% FBS, 2 mM L-glutamine, 100 U/mL P/S, 100 ng/mL MCSF//RANKL (primary) and 30 ng/mL RANKL (cell line). Osteoclasts were determined by tartrate-resistant acid phosphatase (TRAP) staining (Leukocyte TRAP kit; Sigma-Aldrich, St-Louis, MO, USA) following the supplier’s protocol. Bone resorption was assessed in Osteo Assay 96-well plates (Corning, Corning, NY, USA) as described previously [41] and quantified with ImageJ software (https://imagej.nih.gov/ij/).

### 4.6. RNA Extraction, cDNA Synthesis and Real-Time PCR

RNA was extracted using the RNeasy Mini kit (Qiagen, Hilden, Germany) according to the supplier’s protocol. Isolated RNA samples were subjected to DNaseI (Roche) digestion prior to determination of the purity and concentration on a ND-1000 spectrophotometer (Thermo Scientific, Waltham, MA, USA). cDNA synthesis was performed on 100 ng RNA with random hexamer primers using the Transcriptor First Strand cDNA Synthesis Kit (Roche) according to the supplier’s protocol. Real-time PCR (qPCR) was performed on a Lightcycler 480 instrument (Roche) using Kapa SYBR Fast qPCR master mix (Kapa Biosystems (Roche), Basel, Switzerland) using 250 nmol/L of the appropriate primers (Supplementary Table S1). Gene expression was normalised to β-actin and β2-microglubulin expression [42]. All primers were synthesised by Integrated DNA Technologies (Leuven, Belgium). Measurements were performed at least in triplicate. To compare expression levels between different conditions the µCt method was used.

### 4.7. Western Blotting

Cells were lysed in RIPA Lysis and Extraction buffer (Thermo Scientific) supplemented with Complete Protease Inhibitor Cocktail (Roche). 20 µg of proteins were separated by gel electrophoresis on a 10% SDS-polyacrylamide gel and transferred onto PVDF membranes (BioRad, Hercules, CA, USA). Membranes were blocked with 5% BSA/PBS/Tween20 and incubated overnight at 4 °C with primary antibodies (gal-1: R&D Systems, Minneapolis, MN, USA; α-tubulin: Sigma-Aldrich, St-Louis, MO, USA). The next day, blots were incubated with HRP-conjugated secondary antibodies (Dako (Agilent), Santa Clara, CA, USA) followed by visualization on an ImageQuant LAS4000 (GE Healthcare, Chicago, IL, USA).

### 4.8. Immunofluorescence

Cells were cultured on coverslips and fixed with 4% paraformaldehyde for 15 min at room temperature (RT). Fixed cells were washed with PBS and permeabilised with 1% Triton X-100 in PBS for 10 minutes. After washing with PBS, cells were blocked with 5% BSA/PBS and subsequently incubated with gal-1 antibody (R&D Systems) diluted in 5% BSA/PBS for 30 min at RT. Cells were then washed with PBS, followed by incubation with secondary antibody (R&D Systems) and simultaneously phalloidin-FITC (Sigma-Aldrich) for 30 minutes at RT. After washing, nuclei were coloured with DAPI (Sigma-Aldrich) for 5 min at RT and then coverslips were mounted in mowiol mounting solution. Cells were examined under A1R confocal microscopy (Nikon, Tokyo, Japan).

### 4.9. Mice Studies

C57BL/6 wild-type were bred at our animal facility. Gal-1 knock-out (*Lgals1^−/−^*) mice were a gift from Dr. Françoise Poirier (Institut Jacques Monod, Université Paris Diderot, Paris, France). Both strains were kept in specific pathogen-free conditions and water was supplied ad libitum. All animal procedures were approved by the ethical committee. Ethical committee name: Commission Ethique Animale—Université de Liège; approval code: # 14-1635 (given on 24 December 2014). Prior to bone marrow transplantation, eight to twelve-week mice were irradiated at 6Gy. Two hours post-irradiation, 2 × 10^6^ bone marrow cells from syngeneic mice were injected via tail vein injection. Hematopoietic stem cells from gal-1^−/−^ mice were injected in recipient gal-1^−/−^ and hematopoietic stem cells from WT mice injected in WT mice. 24 h after transplantation, 2 × 10^6^ 5TGM.1 GFP+ cells were inoculated intravenously. During the experiments with bisphosphonates, mice received either PBS (control group; Lonza) or 1.5 mg/kg pamidronate (Sigma-Aldrich) subcutaneously once per week. Bone marrow infiltration of MM cells in mice was determined by FACS detection of GFP+ cells on a FACSCantoII flow cytometer (BD Biosciences, Franklin Lakes, NJ, USA). All experimental procedures were approved by the University of Liege (Liège, Belgium) Ethical Committee.

### 4.10. Micro-Computed Tomography

Micro-computed tomography (µCT) was performed on distal femurs from age- and sex-matched mice with the Skyscan 1172 system (Bruker, Billerica, MA, USA) as described previously [41]. Bone parameters were calculated using CTAn software (Bruker). 3D models of bones were generated using CTVol software (Bruker).

### 4.11. Statistical Analysis

All in vitro experiments were performed in triplicate. Results are shown as means +/− standard error and representative pictures are shown. For comparisons of 2 means, a Student t-test was used. For comparisons of multiple means, a one-way ANOVA was used, followed by a Tukey’s post-hoc test. All statistical analyses were performed with Prism 5 software (GraphPad software, San Diego, CA, USA). *p*-values below 0.05 were considered significant and *p*-values are represented as follows: * *p* < 0.05, ** *p* < 0.01, *** *p* < 0.001.

## 5. Conclusions

In summary, the current study suggests that a low expression level of galectin-1 potentiates resorptive function. Additionally, a total lack of gal-1 in the bone microenvironment allows faster development of multiple myeloma bone disease, supporting a role of gal-1 in the bone-marrow microenvironment. These findings extend the knowledge of the implication of glycan-binding proteins in osteoclast biology and multiple myeloma, which is essential in further developing therapeutic strategies for the treatment of multiple myeloma bone disease.

## Supplementary Materials

The following are available online at https://www.mdpi.com/2072-6694/11/2/261/s1, Figure S1: Bone µCT analysis of C57BL/6 WT and gal-1^−/−^ MM-bearing mice, Figure S2. Pamidronate treatment of C57BL/6 gal-1^−/−^ MM-bearing mice, Figure S3. Western blotting analysis of galectin-1 in monocytes (MO) and osteoclasts (OCLs), Table S1. Real-time primer sequences.
