# Supplementary material for: Loss of Stromal Galectin-1 Enhances Multiple Myeloma Development: Emphasis on a Role in Osteoclasts
Source: Cancers (Basel). 2019 Feb 23;11(2):261. doi: 10.3390/cancers11020261 (PMC6406775; doi:10.3390/cancers11020261)
Supplement: Supplementary file 1 [file cancers-11-00261-s001.pdf]

Supplementary Materials: Loss of Stromal Galectin-1 Enhances Multiple Myeloma Development: Emphasis on a Role in Osteoclasts

Joséphine Muller, Elodie Duray, Margaux Lejeune, Sophie Dubois, Erwan Plougonven, Angélique Léonard, Paola Storti, Nicola Giuliani, Martine Cohen-Solal, Ute Hempel, Victor L. Thijssen, Yves Beguin, Roy Heusschen and Jo Caers

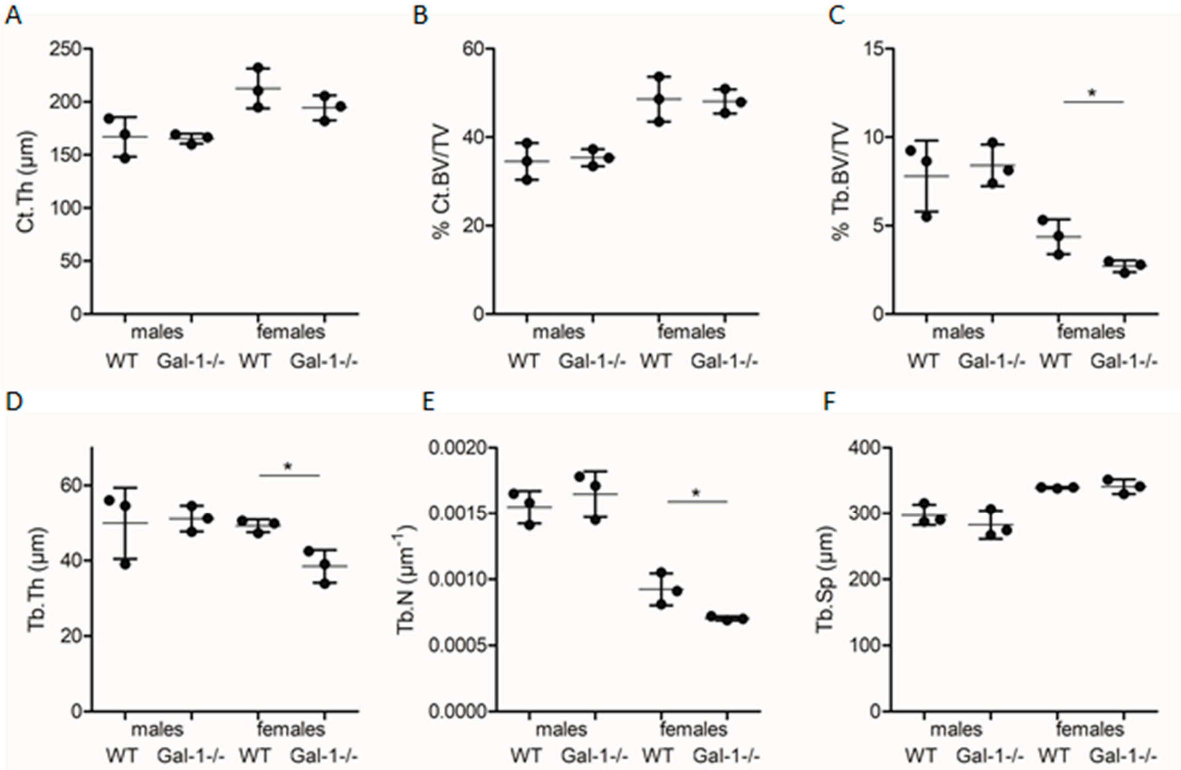

Figure S1. Bone µCT analysis of C57BL/6 WT and gal-1$^{-/-}$ MM-bearing mice. (**A**) Cortical thickness (**B**) Cortical bone volume (**C**) Trabecular bone volume (**D**) Trabecular thickness (**E**) Trabecular number (**F**) Trabecular separation. Data shown are the mean ± standard error of three mice, and all results shown are representative of three independent experiments. * $p < 0.05$.

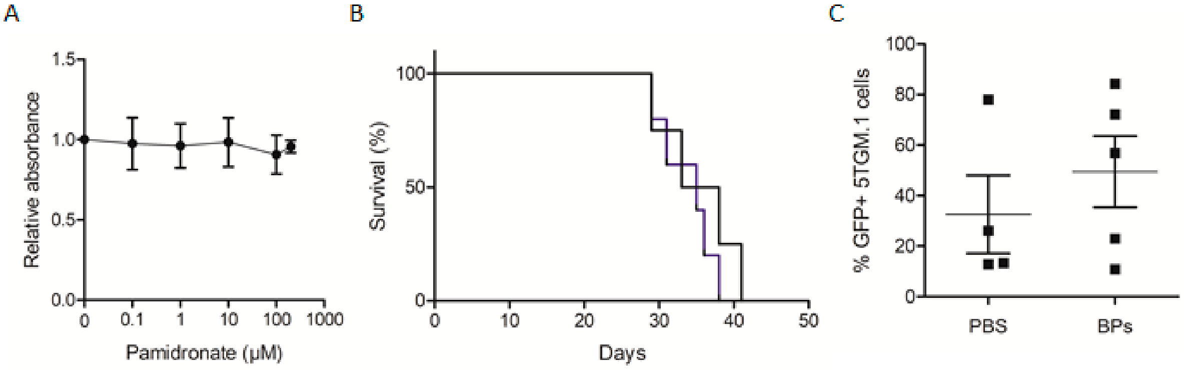

Figure S2. Pamidronate treatment of C57BL/6 gal-1$^{-/-}$ MM-bearing mice. (**A**) Proliferation assay of pamidronate on 5TGM.1 cells. (**B**) Survival curve of gal-1$^{-/-}$ MM-bearing mice treated with BPs (blue line) vs. PBS (black line). (**C**) 5TGM.1 GFP+ bone marrow infiltration of PBS/BPs gal-1$^{-/-}$ MM-bearing mice. Data (in A and C) are represented as mean +/− standard error. All results shown are representative of three ($n = 3$) independent experiments.

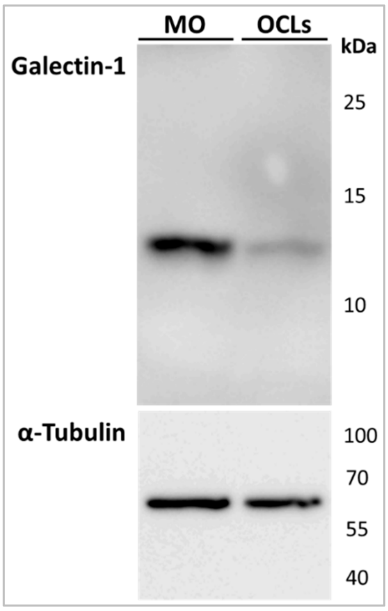

Figure S3. Western blotting analysis of galectin-1 in monocytes (MO) and osteoclasts (OCLs). Galectin-1 monomer expected around 14kDa; α-tubulin, used as an endogenous control, expected around 55kDa. One representative western blot out of three ($n = 3$) independent experiments is shown.

Table S1. Real-time primer sequences.

Gene	Forward	Reverse
Galectin-1	TGTGTGTAACACCAAGGAAGAT	ACCTCTGTGATGCTCCCG
Osteoclast function		
NFATc1	TGAGGCTGGTCTTCCGAGTT	CGCTGGGAACACTCGATAGG
Cathepsin K	CAGCAGAGGTGTGTACTATG	GCGTTGTTCTTATTCCGAGC
TRAP	TCCTGGCTCAAAAAGCAGTT	ACATAGCCCACACCGTTCTC
Calcitonin R	CTTCCATGCTGATCTTCTGG	CAGATCTCCATTGGGCACAA
Integrin αv	GGGCCTATTGTTCAGCACAT	GATTCCACAGCCCAAAGTGT
Integrin β3	GTAATCGAGATGCCCCAGAG	CTTCCATCCAGGGCAATATG
References		
β-actin	TCTGGCTCCTAGCACCATG	AAAACGCAGCTCAGTAACAG
β2MG	GCTACGTAACACAGTTCCAC	TGATGCTTGATCACATGTCTCG

© 2019 by the authors. Licensee MDPI, Basel, Switzerland. This article is an open access article distributed under the terms and conditions of the Creative Commons Attribution (CC BY) license (http://creativecommons.org/licenses/by/4.0/).
